# Supplementary material for: Respiratory cryptosporidiosis in Malawian children with diarrheal disease
Source: PLoS Negl Trop Dis. 2021 Jul 30;15(7):e0009643. doi: 10.1371/journal.pntd.0009643 (PMC8357119; doi:10.1371/journal.pntd.0009643)
Supplement: S1 Table — (DOCX) [file pntd.0009643.s001.docx]

**S1 Table.** Full characteristics of study population at enrollment

| Characteristic | Children with diarrhea (n=162) | Cryptosporidium detected (n=37) | | Cryptosporidium not detected (n=123) | P value |
| --- | --- | --- | --- | --- | --- |
| **Demographics** |  |  | |  |  |
| Father is employed (%) | 150 (93%) | 33 (94%) | | 114 (92%) | 0.742 |
| Highest level of maternal education | | | | | |
| Did not complete any schooling | 62 (38%) | 15 (41%) | | 46(37%) | 0.829 |
| Completed primary school | 74 (46%) | 16 (43%) | | 57 (46%) |  |
| Completed secondary school and above | 26 (16%) | 6 (16%) | | 20 (16%) |  |
| Number of household members (SD) | 4.5 (1.4) | 4.1 (1.2) | | 4.6 (1.4) | 0.061 |
| Adults ≥18 years | 2.1 (0.8) | 1.9 (0.5) | | 2.1 (0.8) | 0.072 |
| Children <18 years | 2.3 (1.1) | 2.1 (1.1) | | 2.3 (1.1) | 0.322 |
| Children <5 years | 1.2 (0.5) | 1.2 (0.4) | | 1.2 (0.6) | 0.107 |
| Number of people living/sleeping regularly in the compound for the past 6 months (SD) | 4.4 (1.4) | 4.1 (1.2) | | 4.5 (1.4) | 0.094 |
| Other household members with respiratory symptoms | 46 (28%) | 14 (38%) | | 32 (26%) | 0.236 |
| Other household members with GI symptoms | 13 (8%) | 4 (11%) | | 9 (7%) | 0.487 |
| Shared pit latrine/toilet for disposal of feces (%) |  |  | |  |  |
| 2 households | 114 (70%) | 27 (75%) | | 84 (69%) | 0.442 |
| 3-5 households | 45 (28%) | 8 (22%) | | 37 (30%) |  |
| ≥6 households | 3 (2%) | 1 (3%) | | 1 (1%) |  |
| Residential animals in the compound (%) | 107 (66%) | 22 (59%) | | 84 (68%) | 0.359 |
| Goat | 6/107 (6%) | 2 (5%) | | 4 (5%) | 0.345 |
| Cow | 0 | - | | - | - |
| Pig | 2 (2%) | 1 (5%) | | 1 (1%) | 0.362 |
| Fowl | 31 (29%) | 7 (33%) | | 24 (29%) | 0.669 |
| Dog | 31 (29%) | 4 (19%) | | 26 (31%) | 0.280 |
| Cat | 17 (16%) | 5 (24%) | | 11 (13%) | 0.222 |
| Rodents | 72 (67%) | 13 (62%) | | 59 (70%) | 0.462 |
| **Child health indicators** |  |  | |  |  |
| Breastfeeding status (%) |  |  | |  |  |
| No breastfeeding | 22 (14%) | 5 (14%) | | 17 (14%) | 1.000 |
| Partial breastfeeding | 134 (83%) | 31 (84%) | | 101 (82%) |  |
| Exclusive breastfeeding | 6 (4%) | 1 (3%) | | 5 (4%) |  |
| Given intravenous fluids in 7 days before admission | 63 (61%) | 17 (46%) | | 45 (37%) | 0.696 |
| Used antibiotics in 7 days before admission | 99 (61%) | 21 (57%) | | 76 (62%) | 0.699 |
| Referred to a health facility for a diarrhea episode in the past 7 days | 136 (84%) | 34 (92%) | | 101 (82%) | 0.292 |
| **Water source** |  |  | |  |  |
| Drinking (%) |  |  |  | |  |
| Piped water | 126 (78%) | 28 (80%) | 95 (77%) | | 0.728 |
| Well/borehole | 36 (22%) | 7 (20%) | | 28 (23%) |  |
| Pond/lake, river/stream | 0 | 0 | | 0 |  |
| Cooking (%) |  |  |  | |  |
| Piped water | 122 (75%) | 28 (78%) | 91 (75%) | | 0.776 |
| Well/borehole | 40 (25%) | 8 (22%) | 31 (25%) | |  |
| Pond/lake, river/stream | 0 | 0 | | 0 |  |
| Bathing water (%) |  |  |  | |  |
| Piped water | 101 (62%) | 23 (64%) | 75 (62%) | | 0.508 |
| Well/borehole | 53 (33%) | 10 (28%) | | 42 (34%) |  |
| Pond/lake, river/stream | 8 (5%) | 3 (8%) | | 5 (5%) |  |
| Utensil water (%) |  |  | |  |  |
| Piped water | 112 (69%) | 23 (66%) | | 86 (70%) | 0.073 |
| Well/borehole | 48 (30%) | 10 (29%) | | 37 (30%) |  |
| Pond/lake, river/stream | 2 (1%) | 2 (6%) | | 0 |  |
| Washing water (%) |  |  |  | |  |
| Piped water | 82 (50%) | 16 (46%) | 65 (53%) | | 0.447 |
| Well/borehole | 41 (25%) | 8 (23%) | | 32 (26%) |  |
| Pond/lake, river/stream | 39 (24%) | 11 (31%) | | 26 (21%) |  |
| Time taken to get to water and return, minutes (SD) | 26.9 (14.1) | 26 (13.8) | | 27.1 (14.1) | 0.708 |
| Number of trips made to fetch water per week (SD) | 27.1 (10) | 25 (7.9) | | 27.7 (10.4) | 0.192 |
| Treated water for drinking^a^ | 23 (14%) | 6 (17%) | | 17 (14%) | 0.596 |
| Child given untreated drinking water in the last week before admission | 74 (46%) | 15 (43%) | | 57 (46%) | 0.715 |

^a^Boiling, or using cloth chlorine, ceramic or other filters.
