# Supplementary material for: Respiratory cryptosporidiosis in Malawian children with diarrheal disease
Source: PLoS Negl Trop Dis. 2021 Jul 30;15(7):e0009643. doi: 10.1371/journal.pntd.0009643 (PMC8357119; doi:10.1371/journal.pntd.0009643)
Supplement: S2 Table — (DOCX) [file pntd.0009643.s002.docx]

**S2 Table.** *Cryptosporidium-*positive at enrollment study population characteristics, stratified by age

|  | <12 months (n=24) | 12-24 months (n=13) | P value |
| --- | --- | --- | --- |
| **Demographics and indicators** |  |  |  |
| Male sex (%) | 17 (70%) | 7 (54%) | 0.301 |
| Child HIV status (%)  Positive  Unknown | 0  19 (79%) | 2 (15%)  5 (38%) | 0.022 |
| HAZ (SD) | -1.2 (2.4) | -1.7 (1.4) | 0.561 |
| WAZ (SD) | -0.9 (1.5) | -1.0 (0.9) | 0.717 |
| WHZ (SD) | 0.0( 1.8) | -0.4 (1.1) | 0.519 |
| **Symptoms** |  |  |  |
| Respiratory symptoms in past 7 days | 16 (67%) | 10 (77%) | 0.515 |
| Vomiting in past 7 days | 13 (54%) | 5 (38%) | 0.362 |
| Abdominal pain in last 7 days | 8 (33%) | 5 (38%) | 0.755 |
| **Cryptosporidium detection** |  |  |  |
| Detection in NP | N=1 | N=3 |  |
| NP Ct values (SD) | 32.9 | 33.7 (1.2) | - |
| Detection in sputum | N=6 | N=5 |  |
| Sputum Ct values (SD) | 31.8 (2.6) | 30.9 (4.2) | 0.657 |
| Detection in stool | N=23 | N=13 |  |
| Stool Ct values (SD) | 27.5 (4.4) | 30.2 (3.5) | 0.072 |
| Detected in respiratory tract only | 1 (4%) | 0 | 0.637 |
| Detected in GI tract only | 18 (75%) | 8 (62%) |  |
| Detected in respiratory and GI tract | 5 (21%) | 5 (38%) |  |
| **Outcome** |  |  |  |
| Mortality | 1 (4%) | - | - |

Ct, cycle threshold; HAZ, height-for-age z score; NP, nasopharynx; SD, standard deviation; WAZ, weight-for-age z score; WHZ, weight-for-height z score
