# Supplementary material for: Respiratory cryptosporidiosis in Malawian children with diarrheal disease
Source: PLoS Negl Trop Dis. 2021 Jul 30;15(7):e0009643. doi: 10.1371/journal.pntd.0009643 (PMC8357119; doi:10.1371/journal.pntd.0009643)
Supplement: Attachment — Submitted filename: IrohTam_2021_RespiratoryCryptoMalawi_reviewer_comments.pdf [file pntd.0009643.s003.pdf]

**Iroh Tam et al. 2021 Respiratory Crypto Malawi review**

*Cryptosporidium* is most well known as a gastrointestinal parasite and its ability to invade the respiratory tract is poorly understood. This study carefully characterizes both gastrointestinal and respiratory symptoms of children hospitalized with diarrhea and is a welcome advance in the field. It is notable for the longitudinal design that is an improvement over previous studies. Assuming the comments below are addressed satisfactorily, I would support publication of this manuscript.

**Specific comments**

Throughout: *Cryptosporidium* should be capitalized and italicized

Line 37: The number and percentage of participants with mild-moderate malnutrition seems to be missing.

Lines 120, 129, 132: suggest clarifying what species of *Cryptosporidium* are detected with the primers used, e.g., *hominis*, *parvum*, etc. The specific primers used should be documented to enable potential comparison with Ct values obtained by other labs.

Line 135: I suggest adding a list of what respiratory co-pathogens were tested for here. I tried to look up this information on the manufacturer's website, but it seems the company has been acquired and it was not straightforward to find.

Line 154: It would be helpful to understand the overlap of subjects positive in either sputum and/or NP samples at enrollment. Were there some with only sputum and only NP? Or were all NP-positive subjects also sputum-positive? Same questions for the 2 subjects that were stool-negative at enrollment.

Line 189-190: Some additional details about respiratory co-pathogens would be useful here, particularly TB screening data. How did the Ct levels for co-pathogens compare with Cts for Crypto? Given the high frequency of co-pathogens, it raises a question about the correlation of respiratory symptoms with Crypto-positive sputum/NP samples. It seems there may be many cases where respiratory symptoms could equally be attributed to co-pathogens other than Crypto. I recognize that respiratory co-pathogens were not evaluated in enrollment samples and that the authors acknowledge this limitation in the Discussion.

Line 242: The acronym "QECH" should be spelled out.

Line 259: I suggest referring to reference #8 as "Uganda study" rather than "Mor study" for consistency and clarity.

Line 499: Please clarify how the patients shown were selected.

Table 1

There appears to be an error on the line “Residential animals in the compound (%)” under the heading of *Cryptosporidium* positive subjects: the figure is listed as “21 (20%)”, however 21 out of 36 subjects would be 58.3%, not 20%.

Footnote a [“Values are mean (SD)”] is unnecessary as “mean (SD)” is already indicated in the description of the referenced line.

The line “Unreadable” under “Sputum quality (%)” is marked with footnote c, but there is no footnote c at the bottom of the table.

Footnote b is missing.

Supplementary table 1:

The title of the line “Shared pit latrine/toilet for disposable of feces (%)” should read “…disposal…”.

There seems to be an error in the footnotes of this table. Footnote a (“Boiling, or using cloth chlorine, ceramic or other filters”) seems to apply to the line “Treated water for drinking”, which is marked with footnote b. On the other hand, footnote b (“Unable to assess sputum quality due to poor appearance”) seems like it does not apply to this table as there is no data related to sputum collection. This also begs the question of what is the correct footnote for the line, “Time taken to get to water and return, minutes”, as this is marked with footnote a.

Figures 1 and 2

I suggest inverting the y-axes so that lower Ct values corresponding to greater amounts of parasite RNA detected are higher up on the chart.

Figure 2

I suggest narrowing the y-axis range from 0- 40 to 20-40 (for example), to increase resolution of charts. I also suggest plotting each graph in a portrait rather than landscape aspect ratio to accentuate the changes in Ct values over time.
