# Supplementary material for: Respiratory cryptosporidiosis in Malawian children with diarrheal disease
Source: PLoS Negl Trop Dis. 2021 Jul 30;15(7):e0009643. doi: 10.1371/journal.pntd.0009643 (PMC8357119; doi:10.1371/journal.pntd.0009643)
Supplement: Attachment — Submitted filename: PNTD-D-21-00656_rev.docx [file pntd.0009643.s004.docx]

This study addresses whether respiratory involvement of cryptosporidium is a transient phenomenon in diarrheal disease or a reservoir for gastrointestinal disease. Previous studies have screened for cryptosporidium in sputum but have not determined whether respiratory involvement is a transient phenomenon or a reservoir for gastrointestinal (GI) disease. Malawian children aged 2-24 months and presenting with primary GI symptoms in Queen Elizabeth Central Hospital in Blantyre were screened for cryptosporidium in collected NP swab, induced sputum, and stool sample using PCR. A total of 162 were recruited into the study from March 2019 to April 2020. Children who were cryptosporidium-positive on any one of NP/sputum/stool were then visited every two weeks for evaluation of symptoms, physical assessment, as well as NP/sputum/stool sampling for a total of 8 weeks.

Thirty-six of 162 children were PCR-positive for cryptosporidium at enrollment (34 stool, 11 sputum, and 4 NP). A greater proportion of children positive for cryptosporidium in both the respiratory and GI tract over this period reported respiratory and GI symptoms compared to those children with only GI detection. These children also had longer GI shedding. These differences in symptoms and shedding were not significant.

These results potentially are important since it suggests that the respiratory tract is a significant reservoir for cryptosporidium infection in addition to the GI tract. As the authors note this suggests that therapeutic drug development may require a drug that targets the lungs as well as the GI tract to cure children.

There are a number of issues that need to be considered. Foremost is the small sample of children who were positive and followed for the 8 weeks. Only identifying 36 children who were positive for cryptosporidium in one of the stool, nasal or sputum samples may be the reason why the comparative differences in the outcomes are not significant in Table 3. This small sample may be partly due to the onset of the Covid-19 in Malawi, which did not allow further enrollment and follow-up of children and so beyond the control of the researchers. This problem is not offset by the longitudinal observational design. The authors have been able to describe the dynamics of respiratory cryptosporidium among children with diarrheal disease, which is commendable, but at this point, these are preliminary results at least for the longitudinal follow-up.

The results presented in some of the tables and figures could be presented more concisely. Table 1 provides a lot of information about the characteristics of the enrolled children. This could be reduced especially since no significant differences were found between cryptosporidium infected children and non-infected children. Some of the numbers presented in table 1 are not correct, specifically the percentage of households with animals in the compound is wrong.

Could you stratify some of these comparisons by age or gender? For example, the authors might consider exploring these patterns by age comparing the dynamics among children who were < 1 year to the dynamics among children < 2 years. The sample although still somewhat small may allow the identification of differences.

I think the manner in which the results are presented in Figure 2 is not informative. There are too many panels and so it is hard to see any consistent patterns. Can these be presented more concisely as well?

Abbreviations should be used for height-for age (HAZ), weight-for-age (WAZ) and weight-for-height (WHZ) throughout the text.

Overall, the results concerning the dynamics of respiratory and GI cryptosporidium infections are interesting and important. The small sample size makes it difficult to arrive at definitive conclusion and so the authors should stress that in their conclusions.
