# Supplementary material for: Respiratory cryptosporidiosis in Malawian children with diarrheal disease
Source: PLoS Negl Trop Dis. 2021 Jul 30;15(7):e0009643. doi: 10.1371/journal.pntd.0009643 (PMC8357119; doi:10.1371/journal.pntd.0009643)
Supplement: Attachment — Submitted filename: CFR_cover letter_response.docx [file pntd.0009643.s005.docx]

# Malawi-Liverpool-Wellcome Trust

# Clinical Research Programme

P.O Box 30096, Chichiri, Blantyre 3, Malawi.

Tel. +265 1 876444 Fax +265 1 875774

Jong-Yil Chai

Associate Editor, *PLoS NTD*

5 July 2021

Dear Dr. Chai,

We thank the reviewers for their constructive and helpful feedback on our original article titled ‘**Respiratory cryptosporidiosis in Malawian children with diarrheal disease.**’ We have incorporated their comments and are very pleased to submit our revised manuscript with tracked changes as well as our detailed response to reviewers. The review comments sent as attachments were not labelled as to which reviewer they were from – therefore, we apologise if we attributed the review comments to the wrong person. Both tracked changes and a clean version are attached.

Our response to reviewer comments are below:

**Reviewer #1:**

*Cryptosporidium* is most well known as a gastrointestinal parasite and its ability to invade the respiratory tract is poorly understood. This study carefully characterizes both gastrointestinal and respiratory symptoms of children hospitalized with diarrhea and is a welcome advance in the field. It is notable for the longitudinal design that is an improvement over previous studies. Assuming the comments below are addressed satisfactorily, I would support publication of this manuscript.

**Author comments:** Many thanks for the comment, we agree that these findings are a contribution to the literature.

**Specific comments**

Throughout: *Cryptosporidium* should be capitalized and italicized

**Author comments:** Changes made.

Line 37: The number and percentage of participants with mild-moderate malnutrition seems to be missing.

**Author comments:** Mean HAZ values (-1.6 (SD 2.1)) were included (L38).

Lines 120, 129, 132: suggest clarifying what species of *Cryptosporidium* are detected with the primers used, e.g., *hominis*, *parvum*, etc. The specific primers used should be documented to enable potential comparison with Ct values obtained by other labs.

**Author comments:** The primer-probe set used to detect the *Cryptosporidium* 18s gene is a qPCR adaptation of a previously employed set (Stroup et al. 2006) and detects all human-pathogenic Cryptosporidium species. We have included a reference to the specific primers used (L128).

Line 135: I suggest adding a list of what respiratory co-pathogens were tested for here. I tried to look up this information on the manufacturer’s website, but it seems the company has been acquired and it was not straightforward to find.

**Author comments:** We included the list of respiratory and stool co-pathogens tested (L133-144).

Line 154: It would be helpful to understand the overlap of subjects positive in either sputum and/or NP samples at enrollment. Were there some with only sputum and only NP? Or were all NP-positive subjects also sputum-positive? Same questions for the 2 subjects that were stool-negative at enrollment.

**Author comments:** The overlap is mentioned in the results (L196). One patient was positive in NP but not sputum; we have included this in the results (L196-199).

Line 189-190: Some additional details about respiratory co-pathogens would be useful here, particularly TB screening data. How did the Ct levels for co-pathogens compare with Cts for Crypto? Given the high frequency of co-pathogens, it raises a question about the correlation of respiratory symptoms with Crypto-positive sputum/NP samples. It seems there may be many cases where respiratory symptoms could equally be attributed to co-pathogens other than Crypto. I recognize that respiratory co-pathogens were not evaluated in enrollment samples and that the authors acknowledge this limitation in the Discussion.

**Author comments:** We agree with the reviewer that the high frequency of co-pathogens may confound the respiratory symptoms documented, and have noted this in the discussion (L325). We considered including additional information on respiratory co-pathogen Ct counts. However, the focus of this paper was on dynamics of cryptosporidium specifically, and we felt further focus on the respiratory co-pathogens – aside from noting the high frequency and potential confounding – was outside the scope of this paper. These details are being examined in detail in a separate paper.

Line 242: The acronym “QECH” should be spelled out.

**Author comments:** This has been done (L96).

Line 259: I suggest referring to reference #8 as “Uganda study” rather than “Mor study” for consistency and clarity.

**Author comments:** This has been done (L270).

Line 499: Please clarify how the patients shown were selected.

**Author comments:** All enrolled participants were included in Table 2. For Table 3, participants with detection at enrollment in GI tract v. GI and respiratory tract comprised the first two groups (Table 3A). The second two groups (Table 3B) comprised participants with detection *at any point* through the study period. We have clarified the language (Table 3, L228, L487)

Table 1

There appears to be an error on the line “Residential animals in the compound (%)” under the heading of *Cryptosporidium* positive subjects: the figure is listed as “21 (20%),” however 21 out of 36 subjects would be 58.3%, not 20%.

**Author comments:** This has been corrected (Table 1). Upon review, we noted some additional inconsistencies that have now been corrected.

Footnote a [“Values are mean (SD)”] is unnecessary as “mean (SD)” is already indicated in the description of the referenced line.

**Author comments:** This has been removed (Table 1).

The line “Unreadable” under “sputum quality (%)” is marked with footnote c, but there is no footnote c at the bottom of the table.

Footnote b is missing.

**Author comments:** These has been corrected (Tables 1-3).

Supplementary table 1:

The title of the line “Shared pit latrine/toilet for disposable of feces (%)” _should read “…disposal…”.

There seems to be an error in the footnotes of this table. Footnote a (“Boiling, or using cloth chlorine, ceramic or other filters”) seems to apply to the line “Treated water for drinking”, which is marked with footnote b. On the other hand, footnote b (“Unable to assess sputum quality due to poor appearance”) seems like it does not apply to this table as there is no data related to sputum collection. This also begs the question of what is the correct footnote for the line “Time taken to get to water and return, minutes”, as this is marked with footnote a.

**Author comments:** Thank you for your keen eye. These has been corrected (Supplementary table 1).

Figures 1 and 2

I suggest inverting the y-axes so that lower Ct values corresponding to greater amounts of parasite RNA detected are higher up on the chart.

**Author comments:** The y axis has been inverted (Figures 1 and 2).

Figure 2

I suggest narrowing the y-axis range from 0- 40 to 20-40 (for example), to increase resolution of charts. I also suggest plotting each graph in a portrait rather than landscape aspect ratio to accentuate the changes in Ct values over time.

**Author comments:** The y axis has been inverted and range narrowed (Figure 2). We changed the graph to portrait orientation.

**Reviewer #2:**

The objectives of the study were clearly explained and a clear testable hypothesis was described. The study design was appropriate for the proposed hypothesis.

The study population was Malawian children aged 2-24 months and presenting with primary GI symptoms in Queen Elizabeth Central Hospital in Blantyre. This population was clearly described and appropriate for the proposed study.

The sample of children who were positive and followed for the 8 weeks was too small to achieve some of the study objectives.

**Author comments:** Thank you for the comments above and we agree the sample size was small. However, the COVID-19 pandemic meant that we were forced to stop our study early and therefore were not able to meet our desired recruitment targets.

The statisitical test could be have been developed more.

**Author comments:** We appreciate the comment and wonder if the reviewer can expand. We note our sample size was small and therefore felt more complex analyses, to include regression analysis, would be improper when the sample size was so small.

There were no concerns about ethical considerations.

The analysis is consistent with the analysis plan and the results are clearly presented.

The results presented in some of the tables and figures could be presented more concisely. Table 1 provides a lot of information about the characteristics of the enrolled children. This could be reduced especially since no significant differences were found between cryptosporidium infected children and non-infected children.

**Author comments:** We have further condensed Table 1 and moved more information to Supplementary Table 1. (Table 1 and Supplementary Table 1).

The authors conclusions are partially supported by the results presented.

**Author comments:** We reviewed the conclusion and have made some edits to ensure the conclusions are supported by the results presented (L759-762).

The limitations regarding the small sample size are not adequately addressed.

**Author comments:** The small sample size was discussed as a limitation in the discussion (L321-323). In addition, we address this again in the conclusion (L337).

The study is innovative and so does increase understanding of results concerning the dynamics of respiratory and GI cryptosporidium infections

**Author comments:** Thank you, we agree the study is innovative.

The public health relevance is addressed by the authors who suggest that therapeutic drug development may require a drug that targets the lungs as well as the GI tract to cure children.

The results presented in some of the tables and figures could be presented more concisely.

**Author comments:** We reviewed and condensed Table 1 and moved information to Supplementary Table 1.

Overall the study in well-executed. The results concerning the dynamics of respiratory and GI cryptosporidium infections are interesting and important. The small sample size makes it difficult to arrive at definitive conclusion and so the authors should stress that in their conclusions.

**Author comments:** Thank you and we agree that the results are interesting and important. We included the statement on small sample size in the conclusion (L337).

I have suggested that the results presented in Table 1 could be stratified by age and or gender to have a better understanding of these dynamics.

**Author comments:** The aim of Table 1 is to provide a breakdown of characteristics by Cryptosporidium positivity. We prepared a separate table stratified by age and include this as Supplementary Table 2 and comment on this in L193-195, although note the numbers are too small to make definitive conclusions.

Associate Editor:

This study addresses whether respiratory involvement of cryptosporidium is a transient phenomenon in diarrheal disease or a reservoir for gastrointestinal disease. Previous studies have screened for cryptosporidium in sputum but have not determined whether respiratory involvement is a transient phenomenon or a reservoir for gastrointestinal (GI) disease. Malawian children aged 2-24 months and presenting with primary GI symptoms in Queen Elizabeth Central Hospital in Blantyre were screened for cryptosporidium in collected NP swab, induced sputum, and stool sample using PCR. A total of 162 were recruited into the study from March 2019 to April 2020. Children who were cryptosporidium-positive on any one of NP/sputum/stool were then visited every two weeks for evaluation of symptoms, physical assessment, as well as NP/sputum/stool sampling for a total of 8 weeks.

Thirty-six of 162 children were PCR-positive for cryptosporidium at enrollment (34 stool, 11 sputum, and 4 NP). A greater proportion of children positive for cryptosporidium in both the respiratory and GI tract over this period reported respiratory and GI symptoms compared to those children with only GI detection. These children also had longer GI shedding. These differences in symptoms and shedding were not significant.

These results potentially are important since it suggests that the respiratory tract is a significant reservoir for cryptosporidium infection in addition to the GI tract. As the authors note this suggests that therapeutic drug development may require a drug that targets the lungs as well as the GI tract to cure children.

There are a number of issues that need to be considered. Foremost is the small sample of children who were positive and followed for the 8 weeks. Only identifying 36 children who were positive for cryptosporidium in one of the stool, nasal or sputum samples may be the reason why the comparative differences in the outcomes are not significant in Table 3. This small sample may be partly due to the onset of the Covid-19 in Malawi, which did not allow further enrollment and follow-up of children and so beyond the control of the researchers. This problem is not offset by the longitudinal observational design. The authors have been able to describe the dynamics of respiratory cryptosporidium among children with diarrheal disease, which is commendable, but at this point, these are preliminary results at least for the longitudinal follow-up.

**Author comments:** We agree that the small sample size limits interpretation of the results. We did include an additional statement on the small sample size in the conclusion (L337).

The results presented in some of the tables and figures could be presented more concisely. Table 1 provides a lot of information about the characteristics of the enrolled children. This could be reduced especially since no significant differences were found between cryptosporidium infected children and non-infected children. Some of the numbers presented in table 1 are not correct, specifically the percentage of households with animals in the compound is wrong.

**Author comments:** We reviewed and condensed Table 1 and moved information to Supplementary Table 1. We also corrected the footnotes and numbers queried.

Could you stratify some of these comparisons by age or gender? For example, the authors might consider exploring these patterns by age comparing the dynamics among children who were < 1 year to the dynamics among children < 2 years. The sample although still somewhat small may allow the identification of differences.

**Author comments:** We stratified by age and include this as a Supplementary Table 2 (L193-195 and Supplementary Table 2).

I think the manner in which the results are presented in Figure 2 is not informative. There are too many panels and so it is hard to see any consistent patterns. Can these be presented more concisely as well?

**Author comments:** We selected fewer graphs and revised the panel to make it smaller.

Abbreviations should be used for height-for age (HAZ), weight-for-age (WAZ) and weight-for-height (WHZ) throughout the text.

**Author comments:** This has been done (L38, Table 1, Table 3, 168, 303, 313).

Overall, the results concerning the dynamics of respiratory and GI cryptosporidium infections are interesting and important. The small sample size makes it difficult to arrive at definitive conclusion and so the authors should stress that in their conclusions.

**Author comments:** We included an additional statement on the small sample size in the conclusion (L337).

We thank the reviewers for their guidance and constructive feedback, and believe the revised manuscript has benefited greatly from the reviewer and editorial comments. We appreciate how constructive the editorial process has been.

We thank you for your consideration.

Yours sincerely,

Pui-Ying Iroh Tam, DMed, FAAP, FPIDS, FIDSA

Senior Paediatrician, Malawi-Liverpool Wellcome Trust Clinical Research Programme

Senior Clinical Lecturer, Liverpool School of Tropical Medicine

Corresponding author: [irohtam@mlw.mw](mailto:irohtam@mlw.mw)
